# Integrated multi-ISE arrays with improved sensitivity, accuracy and precision

**DOI:** 10.1038/srep44771

**Published:** 2017-03-17

**Authors:** Chunling Wang, Hongyan Yuan, Zhijuan Duan, Dan Xiao

**Affiliations:** 1 College of Chemical Engineering, Sichuan University, 29 Wangjiang Road, Chengdu 610064, People’s Republic of China; 2 College of Chemistry, Sichuan University, 29 Wangjiang Road, Chengdu 610064, People’s Republic of China

## Abstract

Increasing use of ion-selective electrodes (ISEs) in the biological and environmental fields has generated demand for high-sensitivity ISEs. However, improving the sensitivities of ISEs remains a challenge because of the limit of the Nernstian slope (59.2/n mV). Here, we present a universal ion detection method using an electronic integrated multi-electrode system (EIMES) that bypasses the Nernstian slope limit of 59.2/n mV, thereby enabling substantial enhancement of the sensitivity of ISEs. The results reveal that the response slope is greatly increased from 57.2 to 1711.3 mV, 57.3 to 564.7 mV and 57.7 to 576.2 mV by electronic integrated 30 Cl^−^ electrodes, 10 F^−^ electrodes and 10 glass pH electrodes, respectively. Thus, a tiny change in the ion concentration can be monitored, and correspondingly, the accuracy and precision are substantially improved. The EIMES is suited for all types of potentiometric sensors and may pave the way for monitoring of various ions with high accuracy and precision because of its high sensitivity.

Ion-selective electrodes (ISEs) have an absolute advantage as ion concentration monitors for their simple equipment, portability, low cost, and good selectivity, and they are widely used for scientific research, clinical diagnostic and environmental monitoring applications^1^,^2^,^3^,^4^,^5^,^6^. Although ISEs are powerful analysis tools in analytical fields, there are still many challenges to address. First, the sensitivities of the ISEs (~59.2/n mV, limited by the Nernstian response slope per decade activity) are not high enough for the analysis of analytes in complex samples such as seawater, estuarine waters, rivers, lakes, and soils^1^. In addition, most ISEs lack the sensitivity required for reporting tiny changes in the sample, which occur in a large number of biological processes^7^,^8^,^9^,^10^,^11^. Moreover, it is widely known that instrumental analysis has rightfully played an overwhelmingly large role in the analytical laboratory as the electronics and computer industries have developed. However, it remains a challenge for instrumental analysis because of the comparatively low accuracy and precision compared with classical analysis methods at high concentration levels^12^. As part of these instrumental analysis methods, many ISEs also cannot provide high precision or stability levels, which limits their applications. Although some quantitative analysis approaches, such as standard addition and the Gran plot method, have been adopted to increase accuracy, they are relatively cumbersome.

Since the first fluoride selective electrode based on LaF_3_ crystals was reported in 1966^13^, the field of ISEs has grown to become quite large, with numerous probes having been developed for the analysis of samples containing many different ions^14^,^15^,^16^,^17^,^18^. The practical applications of ISEs demand high sensitivity as well as high measurement accuracy and precision, which results in the considerable improvement in new membrane materials, new sensing concepts, and new analysis modes. In the past, many research efforts have led to improvements in the lower determination limit or the selectivity of ISEs^19^,^20^,^21^. Although there have been some attempts to increase the sensitivity of ISEs^22^,^23^,^24^,^25^,^26^,^27^,^28^, to the best of our knowledge, a universal detection method that simultaneously enhances the sensitivity and achieves accuracy and precision with a wider working range without complicated operating steps and expensive instruments or agents has not been reported. In this study, we devote our efforts to improving the determination performance of ISEs in terms of the detection method. Here, we propose the EIMES method, which is a universal detection method that is suitable for all types of potentiometric sensors. We hope that this universal detection method can find widespread applications in potentiometric sensors in the future.

Applications for environmental control, especially in the life sciences, have resulted in the inevitable trend of electrode miniaturization. The development of electroactive materials, solid-contact ISEs and sensor membranes^29^,^30^,^31^,^32^,^33^,^34^,^35^,^36^,^37^,^38^,^39^,^40^,^41^,^42^,^43^,^44^ are accelerating the miniaturization of the electrode. In recent years, improvement of the sensitivity via electrodes array has continued to be an area of intense research, particularly in the field of biological and clinical research^45^,^46^,^47^,^48^,^49^. To the best of our knowledge, previous research regarding ISEs arrays has focused on specific electrodes and detection objects, simultaneous detection of multi-component analytes via an array of multiple different electrodes, and the processing of the interference signal between multiple electrodes etc. In many cases, such as in biological and environmental samples, the ion concentration varies only by a few percent, the measurements are very demanding in terms of the requirement of not only potential sensitivity but also potential stability and reproducibility. The aim of our work was to develop a universal EIMES method that provides substantial enhancement of the Nernstian response slope of multi-ISEs. The proposed method is suitable for all types of ISEs and potentiometric-based sensors and can significantly improve the measurement accuracy and precision. Furthermore, we demonstrate the successful application of the EIMES for accurate measurement of a tiny change in ion concentration and potentiometric acid base titration. Some researchers have made good experimental progress in electrode miniaturization to date^50^,^51^,^52^,^53^,^54^,^55^,^56^. With the development of miniaturized electrodes, the EIMES, a sensitive and universal method for accurate measurement, has potential for widespread use.

## Results and Discussion

**Figure 1:**
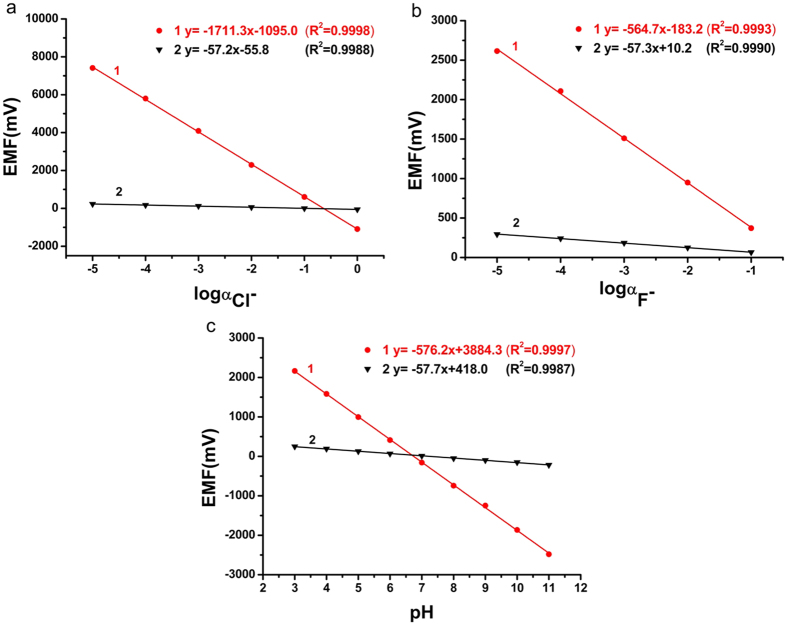
Linear calibration curve obtained by ISEs with changes of Cl^−^, F^−^ and pH. (**a**) Shows a linear fit to the data for the KCl solution with concentration increasing from 1 × 10^−5^ M to 1 M. (**b**) Shows a linear fit to the data for the NaF solution with concentration increasing from 1 × 10^−5^ M to 0.1 M. (**c**) Shows a linear fit to the data for buffer solutions with pH increasing from pH 3 to pH 11. Line 1 in (**a**,**b** and **c**) was obtained using EIMES with 30 Cl^−^ electrodes, 10 F^−^ electrodes and 10 glass pH electrodes, respectively. Line 2 in (**a**,**b** and **c**) was obtained using a single Cl^−^ electrode, F^−^ electrode and glass pH electrode, respectively.

The Nernstian response slope is one of the methods used to evaluate the performance of ISEs. The theoretical slope is 59.2/n (mV) (n is the charge-transfer number of the electrode reaction). It is widely known that a single electrode obeys the response under normal circumstances. In our experiment, the multi-ISEs and a saturated calomel electrode are used as the working electrodes and reference electrode, respectively. The slope of the multi-ISEs based EIMES is much greater than 59.2/n (mV). In this work, a series of F^−^, H^+^ and Cl^−^ concentrations were tested by using an EIMES with ten F^−^ electrodes, ten pH electrodes (EIMES-10) and thirty Cl^−^ electrodes (EIMES-30) as the working electrodes. The results in (Fig. 1a) show that the Nernstian response slope is increased from 57.2 mV for a single Cl^−^ electrode to 1711 mV for EIMES-30 Cl^−^ electrodes per decade activity, which improved the sensitivity by approximately 30-fold. Similarly, EIMES with ten electrodes enhanced the Nernstian response slope by approximately 10-fold over that of a single electrode. Nernstian response slopes are increased from 57.3 mV for a single F^−^ electrode to 564.7 mV for EIMES-10 F^−^ electrodes (Fig. 1b) and from 57.7 mV for a single glass pH electrode to 576.2 mV for EIMES-10 glass pH electrodes (Fig. 1c) per decade activity. The experimental results show that the EIMES detection method can greatly improve the Nernstian response slope, and the slope increases with the increase in the number of electronic integrated electrodes. As a result, we can change the number of ISEs to achieve the ideal measurement sensitivity.

**Figure 2:**
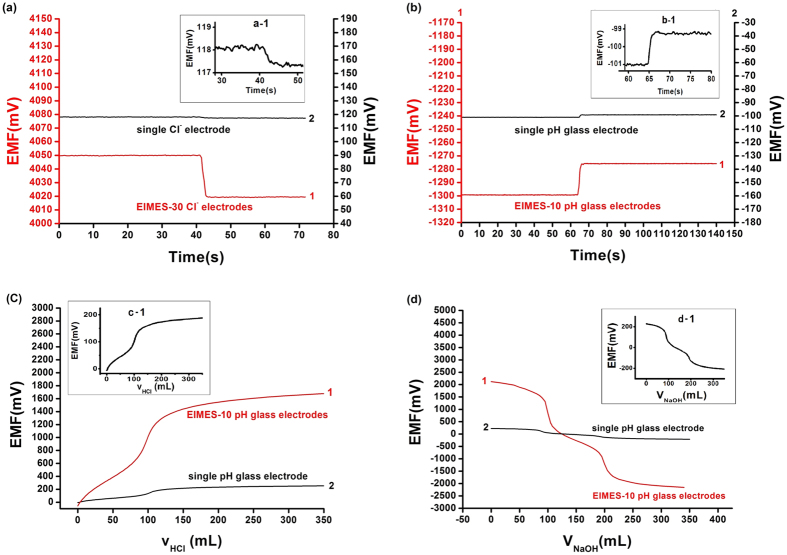
The changes in the potentiometric responses with varying ion concentrations. (**a**) Shows the change in potentiometric responses to Cl^−^ after adding one drop (approximately 0.04 mL) of 0.1 M KCl to 100 mL of 1.0 × 10^−3^ M KCl. Curve 1 and curve 2 in (**a**) were detected by EIMES-30 Cl^−^ electrodes and a single Cl^−^ electrode, respectively. (**b**) Shows the change in potentiometric responses to H^+^ after adding one drop (approximately 0.04 mL) of 1 M HCl to 100 mL buffer solution of pH 9.0. (**c**) Shows the titration curves of histidine obtained by adding 0.001 M HCl solution to 100 mL of 0.001 M histidine solution dropwise. (**d**) Shows the titration curve of H_3_PO_4_ obtained by adding 0.001 M NaOH solution to 100 mL of 0.001 M H_3_PO_4_ solution dropwise. In (**b**,**c** and **d**) curves 1 and curve 2 were detected by EIMES-10 glass pH electrodes and a single glass pH electrode, respectively. The small picture a-1 in the upper of (**a**) is 30 times vertical axis magnification of a single ISE. The small picture b-1, c-1, d-1 in the upper of (**b**,**c**,**d**) respectively, is 10 times vertical axis magnification of a single ISE.

Figure 2a shows the changes in potentiometric responses to Cl^−^ for adding one drop (approximately 0.04 mL) of 0.1 M KCl to 100 mL KCl solution (1.0 × 10^−3^ M). The potentiometric response of the EIMES-30 Cl^−^ electrodes was obviously changed by approximately 30 mV (Fig. 2a, Curve 1). However, there was about 0.8 mV for a single Cl^−^ electrode (Fig. 2a, Curve 2). According to calculations, the theoretical variation value is approximately 29 mV for EIMES with 30 Cl^−^ electrodes and less than 1 mV for a single Cl^−^ electrode. A similar trend is observed in Fig. 2b: with addition of one drop (approximately 0.04 mL) of 1 M HCl to 100 mL of buffer solution (pH 8.9), the potentiometric response had a significant improvement of approximately 24 mV for EIMES-10 glass pH electrodes (Fig. 2b, Curve 1), whereas for a single glass pH electrode, there is a change of approximately 2.0 mV (Fig. 2b, Curve 2). Calculations of the theoretical variation values correspond to approximately 23 mV for EIMES with 10 glass pH electrodes and 2 mV for a single glass pH electrode. When the ion activities vary at the same interval, using the EIMES method, it is easier to obtain data accurately as the voltage changes.

Furthermore, the EIMES was applied to measure the H^+^ concentration of a weak acid, a weak base and an ampholyte analyte, specifically, H_3_PO_4_ and histidine solutions. Figure 2c shows the titration curve of a histidine solution: 0.001 M HCl solution added dropwise to 100 mL of 0.001 M histidine solution. Figure 2d shows the titration curve of H_3_PO_4_ solution: 0.001 M NaOH solution added dropwise to 100 mL of 0.001 M H_3_PO_4_ solution. In Fig. 2c and d, curve 1 and curve 2 were detected by EIMES-10 glass pH electrodes and a single glass pH electrode, respectively. From the results, obvious abrupt changes in titration curve 1 in Fig. 2c and d are clearly observed. However, for the single electrode, the response is relatively weak. This result demonstrated that the EIMES could be applied in the potentiometric measurement of tiny changes; this capability is attributed to the high Nernstian response slope. In contrast, a single electrode performed either poorly or not at all.

**Figure 3:**
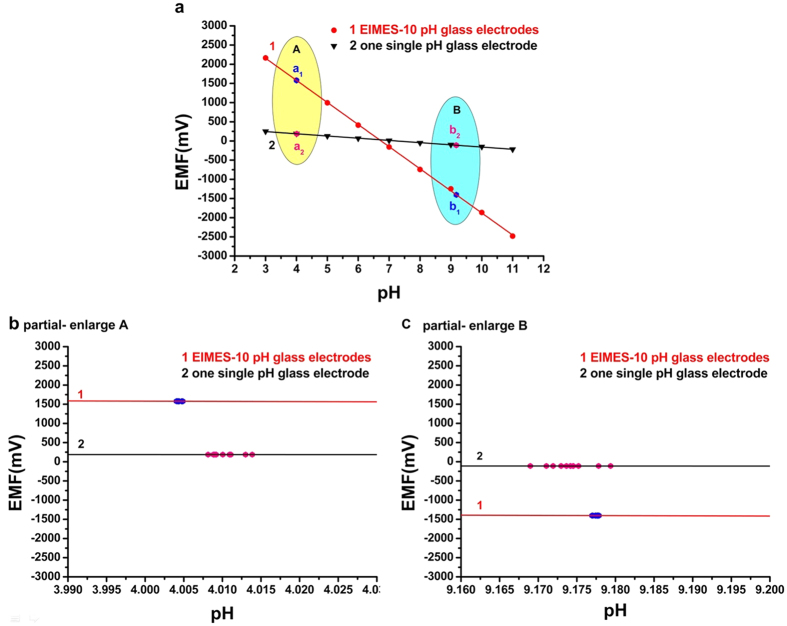
Test experiment using pH 4.003 and pH 9.182 standard buffer solutions. Line 1 and line 2 in Fig. 3 were detected by EIMES-10 glass pH electrodes and a single electrode, respectively. (**b** and **c**) are the partial enlarged views of standard buffer solutions with pH 4.003 and pH 9.182 testing points, respectively. The dots in line 1 were obtained using a single glass pH electrode, and the dots in line 2 were obtained using EIMES-10 glass pH electrodes.

The EIMES detection method represents an advantage in accuracy and precision over the performance of a single electrode. It is known that instrumental analysis has the issue of generally not providing high accuracy or precision at high concentration levels^12^. The EIMES detection method we proposed will hopefully address this issue in the fields of ISE and potentiometric-based sensors. In quantitative analysis, the number of significant figures in a measurement is the number of digits known exactly plus one digit for which the value is uncertain. For example, the Nernstian response slope obtained by a single glass pH electrode is 57.7 mV per decade activity, and the absolute uncertainty is ±0.1 mV; thus, the relative error is approximately ±0.2%. However, for EIMES-10 electrodes of same type at the same condition, the Nernstian response slope is approximately 576.2 mV, which is approximately 10 times greater than that of a single glass pH electrode, and the absolute uncertainty is also ±0.1 mV, but the relative error greatly decreases to approximately ±0.02%. Using EIMES significantly increases the measurement accuracy and precision, as further demonstrated by testing ten separate parallel standard buffers with of pH 9.182 and 4.003 at 25 °C, we obtained average values of 9.17 ± 0.01 and 4.01 ± 0.01, respectively, by a single glass pH electrode; and 9.178 ± 0.001 and 4.004 ± 0.001, respectively, by EIMES-10 glass pH electrodes. The measured values showed that the standard deviation of the EIMES-10 glass pH electrodes is lower than that of a single electrode. It can also be seen from Fig. 3a and the partial enlarged views (Fig. 3b and c) that the data points are distributed more densely when using EIMES (dots in line 1) compared with a single electrode (dots in line 2). Thus, using EIMES yields higher precision than does using a single electrode.

The high sensitivity allows the EIMES method to have high measurement accuracy as well as precision. For an electrochemical cell, The Nernst Equation is as follows:


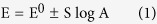


where S represents for the Nernstian response slope and A is the activity of the ion to be measured. According to equation (1), the relative concentration error equation is as follows (for the derivation of the relative concentration error formula, see the Supplementary Equation in the Supplementary Information):


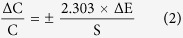


If the potential error (ΔE) remains the same, the higher the response slope, the less the relative concentration error. Take the glass pH electrode as an example. As observed from the linear regression equation in Fig. 1c, the slope of the EIMES-10 glass pH electrodes is 576.2 mV, which is close to a 10-fold increase over that of the single glass pH electrode (57.7 mV). According to equation (2), the relative concentration error of the EIMES-10 glass pH electrodes is close to one-tenth that of the single glass pH electrode. The lower relative error makes the EIMES method more accurate, as demonstrated by two experiments in this work. One experiment involves testing ten parallel samples of the standard buffers of pH 9.182 and 4.003 at 25 °C. The average values determined using the EIMES-10 glass pH electrodes (9.178 and 4.004) are closer to the real values than are those measured using a single glass pH electrode (9.17 and 4.01). The other experiment involves titration: the concentration of the H_3_PO_4_ solution is determined by titrating with a standard NaOH solution. Figure 2d shows the titration curve resulting from adding 0.001 M NaOH solution dropwise to 100 mL of 0.001 M H_3_PO_4_ solution. For the 0.001 M H_3_PO_4_ solution, at the two end points, a sudden change is observed. The theoretical value of the pH at the first endpoint titration (ep1) is 5.267, and that at the second endpoint titration (ep2) is 8.845; these theoretical values were obtained from numerical calculation of the exact formula for a weak polyprotic acid. By taking the derivative of the titration curve, we can obtain the experimental values of 5.283 and 8.859 for EIMES with 10 glass pH electrodes at the first endpoint titration and the second endpoint titration, respectively; however, for a single pH electrode, the experimental values were 5.39 and 8.94, respectively. We can achieve more accurate measurements using the EIMES detection method only one time measurement. The experimental results listed in Table 1 show that lower relative errors and higher accuracy are achieved using the EIMES with 10 pH electrodes than using a single pH electrode. Considering the interference of co-existing ions, we also compared the measurement accuracies of the EIMES and the single electrode in the presence of an interfering ion. Metal ions, such as aluminum ions, interfere with the measurement of fluoride ions by forming a complex with fluoride. Thus, the solutions in TISAB (pH 5.05, no masking reagents) of complexes 1.2 × 10^−4^ M of aluminum at the 5.0 × 10^−4^ M of fluoride level were measured. The measurement had a potential deviation of 128.6 mV using the EIMES-10 F^−^ electrodes and 13.0 mV using a single F^−^ electrode, the relative error are 6.97% and 6.917%. Next, we added 0.1 M sodium citrate as the aluminum masking reagent to the above-mentioned solutions; the potential deviation of 5.4 mV using the EIMES-10 F^−^ electrodes and 2.2 mV using a single F^−^ electrode. The relative errors decreased from 1.18% for the single F^−^ electrode to 0.291% for the EIMES-10 F^−^ electrodes, with the test performed only one time. From these experimental results, the EIMES method is found not to have a significant improvement in detection accuracy in such a co-existing ion situation. This is because that the error is mainly caused by the interfering ion in sample solution rather than the detection method itself. If the interfering ions were eliminated, then the EIMES-multi electrodes method would still provide higher accuracy than the single electrode method.

In addition, compared to a single electrode, the correlation coefficients of the calibration curves obtained from the EIMES with multiple electrodes are increased from 0.9988 to 0.9998 for Cl^−^ electrodes, from 0.9990 to 0.9993 for F^−^ electrodes and from 0.9987 to 0.9997 for glass pH electrodes. Undoubtedly, the accuracy and precision of ion measurement are increased using the EIMES detection method we proposed.

### Components of the EIMES

ISE follows the Nernstian response at normal circumstance, and the theoretical slope is 59.2/n (mV) at 298 K. The Nernstian response of some ISEs made for application in recent researches can reach 58–59/n (mV). There is a report that a super-Nernstian response appears in ISE by adding a pulse current or voltage^22^, which is very rare and can effectively improve the slope of a normal ISE. In this work, an EIMES detection method was developed that allows the Nernstian response slope to be much greater than 59.2/n (mV). In fact, with potentiometric sensors, it is known that for one ISE, the following is valid:


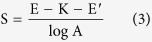


where E is the measured potential within the Nernstian response range, all constant potential contributions of the ISEs themselves are included in K, and E′ is the voltage deviation produced by circuit and the other external conditions.

For multi-ISEs, we have


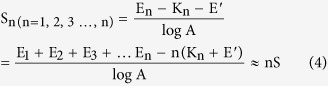


From equation (4), we know that S_n_ is proportional to n. If n is set to 30, then the theoretical slope S_n_ is nearly 30 times that of S, and S improves as the number of electrodes increases.

However, the summation signals of multi-ISEs probably cause noise signal accumulation and invalidate the S_n_. After analyzing the signal components of multi-ISEs, the interference in the slope can be removed by using a suitable determination circuit. The signal f from a single electrode consists of f_1_, f_2_ and f_3_.


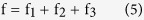


Here, f_1_ is a directive current voltage, represents the effective signal from electrode, whose value is determined by equation (1). The second component, f_2_, is random noise caused by thermal noise in the circuit or induction from the environment. The last component, f_3_, represents a noise component that is called interference noise, which is mainly a periodic signal induced by alternating current because of the high input resistance of the electrode. For f_2_, as random noise, an important characteristic is that it can be decreased to some degree when a sum calculation is applied. For f_3_, although the interference noise can be removed by using a low pass filter, the adoption of a low pass filter would probably cause additional effects on the measurement of the dynamic change in solution concentration. In view of the above factors, a new filter method for electrode signal collection is proposed in this study (more details are presented in the Determination Circuit section).

### Determination Circuit

**Figure 4:**
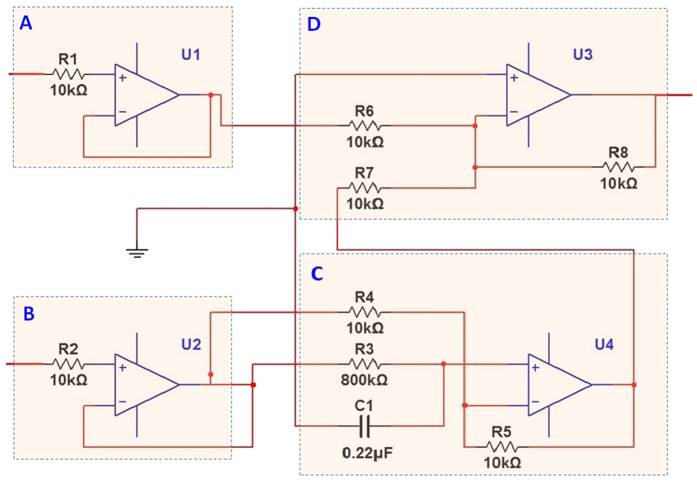
The structure of an input channel unit. The type of the operational amplifiers in Fig. 4 is AD712JN. (**A**) and (**B**) are the signal input terminals of the ISEs. The electrodes are connected two resistors R1 (10 kΩ) and R2 (10 kΩ), and operational amplifiers U1 and U2 are voltage followers. (**C**) Is the phase shift circuit, which is constituted by operational amplifier U4, resistors R3 (800 kΩ), R4 (10 kΩ), and R5 (10 kΩ), and capacitor C1 (0.22 μF). (**D**) Is a two-input adder, which is constituted by operational amplifier U3 and resistors R6 (10 kΩ), R7 (10 kΩ), and R8 (10 kΩ).

The function of the determination circuit is to collect all the signals of the multi-electrode array and take their sum. The total determination circuit contained multiple units, and every unit comprised two input circuits: a phase-shift filter and an adder circuit. The structure of one unit is shown in Fig. 4. Every circuit unit connected two inputs of the working electrodes. Because the output impedance of the glass pH electrode is generally very high, two input circuits formed by two voltage followers (consisted of an operator) were adopted to match the resistance of the electrodes. Next, one of the two-input circuits was connected to a two-input adder (consisted of an operator) directly; the other was followed by the phase-shift filter to remove the interference from the alternating current (AC) in the power supply. Next, the output was sent to the adder.

**Figure 5:**
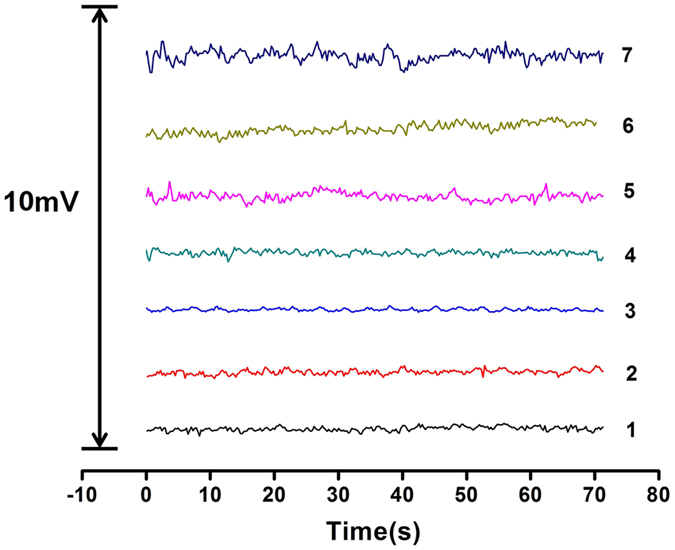
The baseline noise comparison diagram. Curve 1 is the baseline noise diagram of a single Cl^−^ electrode. Curve 2 and 3 are the two Cl^−^ electrodes without and with the phase-shifted filter. Curve 4 and 5 are the ten Cl^−^ electrodes without and with the phase-shifted filter. Curve 6 and 7 are the thirty Cl^−^ electrodes without and with the phase-shifted filter. All the baseline noise diagram were obtained at the same sample solution of 1 × 10^−4^ M Cl^−^.

The reason for adoption of the phase-shift filter was that as an active noise filter, the phase-shift filter is an effective method to suppress interference at a specific frequency without too much interference to the other signal components. For glass pH electrodes, the main interference is the induced electromagnetic noise from the surrounding AC circuit, which is at 50 Hz and can overlap the Nernstian potential signal, resulting in a measurement error. Although the low pass filter can remove this interference, because of the bandwidth and transition band of such a filter, the filter probably also affects the lower frequency components in the dynamic measurement, which are probably contained in the varying useful signal. After the phase-shift filter, the phase of a periodic signal of approximately 50 Hz will be shifted 180°. By adding a periodic signal that has an inverted phase, the output is zero. The circuit of the phase-shift filter is demonstrated in Fig. 4, and it consists of an operator and a resistance-capacity (RC) circuit. Figure 5 shows the baseline noise measurement of a single Cl^−^ electrode (curve 1); two Cl^−^ electrodes without (curve 2) and with the phase-shifted filter (curve 3); ten Cl^−^ electrodes without (curve 4) and with the phase-shifted filter (curve 5); thirty Cl^−^ electrodes without (curve 6) and with the phase-shifted filter (curve 7), respectively, both in the same sample solution of 1 × 10^−4^ M Cl^−^. As shown in Fig. 5, the baseline noise increase with the number of electrodes. The noise of thirty electrodes without phase-shifted filter are larger than ten or one electrodes. The baseline noise with phase-shifted filter is obvious lower than that of without phase-shifted filter and single Cl^−^ electrode.

## Conclusions

The EIMES, which was used as a novel measurement method, was successfully developed to improve the measurement performance of ISEs and potentiometric-based sensors. On one hand, it has been demonstrated that the sensitivity of electronic integrated multi-ISEs is significantly improved, and by using the EIMES, the Nernstian response slope improved as the number of electrodes increased. The high Nernstian response slope allows the EIMES to have an advantage in monitoring tiny changes in ion concentration in solutions. Note that ISEs have a certain lifetime, i.e., the measurement performance degrades, resulting in the gradual decrease of the Nernstian response slope until the effective sensitivity of ISEs is affected, thereby limiting their applicability as working electrodes. This problem can be addressed through the EIMES detection method because of its high sensitivity. Thus, the EIMES detection method can extend the service life of ISEs. On the other hand, using EIMES can improve the measurement accuracy through the lower relative error and the higher measurement precision. The EIMES detection method we propose here is aimed at improving the measurement performance by means of arrays and integrated multi-electrodes rather than fundamentally changing the properties of individual electrodes. Thus, the EIMES method will hopefully be applied in all types of ISEs and potentiometric-based sensors as the electrode miniaturization is further developed. The EIMES method enhances sensitivity and provides a high measurement accuracy and precision, representing a universal potentiometric detection method that provides an opportunity to monitor tiny changes in analytes in routine analysis and in complex, challenging samples in scientific research, clinical diagnostics, and environmental monitoring while being suitable for all potentiometric sensors.

## Experimental

### Reagents

Sodium chloride, sodium citrate, citric acid, sodium fluoride, sodium hydroxide, potassium chloride, sodium dihydrogen phosphate dihydrate, sodium phosphate dibasic dodecahydrate, glacial acetic acid, phosphoric acid, histidine, sodium bicarbonate, sodium borate, potassium hydrogen phthalate, hydrochloric acid, monopotassium phosphate and tris-hydroxymethyl aminomethane and aluminum nitrate were all purchased from Kelong Chemical Co., Ltd. (Chengdu China). All chemicals were of analytical grade and were used as received without further purification. All solutions were freshly prepared with triply distilled water.

### Electrochemical Equipment

**Figure 6:**
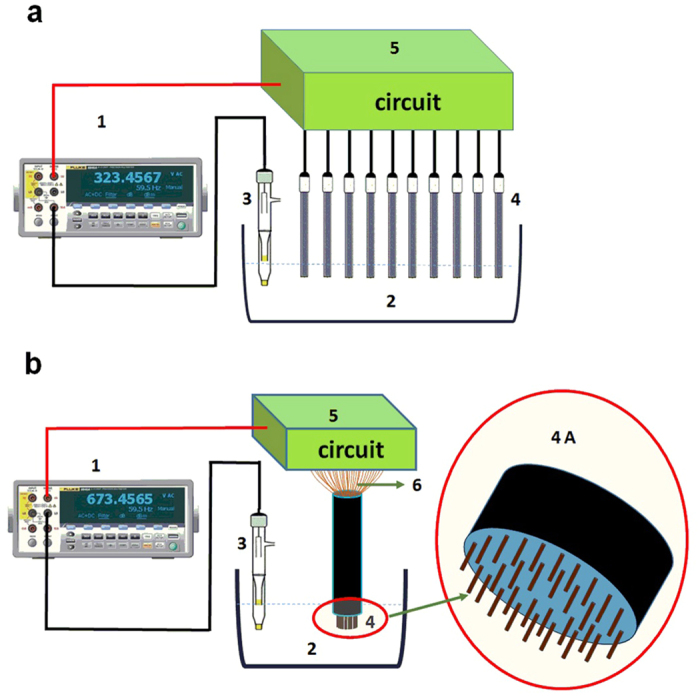
The experimental setup for EIMES with ten commercial ISEs (**a**) and thirty laboratory-made chloride electrodes (**b**). High-precision digital multimeter. 2. Buffer solution. 3. Reference electrode. 4. Multi-commercial ISEs array. 4 A. Enlarged view of the array of thirty chloride electrodes. 5. Functional adder circuit. 6. Copper conductor cable.

A high-precision digital multimeter (Fluke 8846, 6.5 Digit Precision Multimeters) were used to acquire the potentiometric signal of the EIMES. The output signal of EIMES-multielectrodes is the sum of the potentiometric signals, the magnitude of the EIMES exceed the measurement range of commercial ion meters (±1999.9 mV). Thus, the potentiometric signal of the EIMES was obtained using Fluke 8846 that has a maximum measurement range of ±1000 V. In fact, the maximum voltage range in this experiment is from −10 V to + 10 V. A commercial ion meter (Orion SA720) were used only as control to ensure the reliability of EIMES. The comparison results between EIMES-single ISE with Orion SA720 showed that both of them can obtained the same stable digit of 1 mV and consistent measurement results. Glass pH electrodes (231-01), fluoride electrodes (PF-1-01) and a double junction saturated calomel reference electrode (217) were purchased from Shanghai INESA Scientific Instrument Co. Ltd., and chloride electrodes were prepared by anodizing 0.5 mm diameter silver wires in diluted aqueous HCl solution, as previously described^57^. The EIMES was composed of multiple ISEs, a reference electrode and an electronic circuit. The experimental setup of the EIMES is shown in Fig. 6.

### Preparation of F^−^ standard solution

A solution of total ionic strength adjustment buffer (TISAB) was added to the samples to adjust the pH and ionic strength to optimum values for F^−^ measurement. TISAB was prepared according to reference^58^. A standard stock solution (1 M) of F^−^ was prepared by dissolving sodium fluoride in water. Standard solutions of F^−^ were prepared by diluting the stock solution in TISAB to provide a series of concentrations in the range of 1 × 10^−6^ to 0.1 M.

### Preparation of pH standard solution

Standard stock solutions of citric acid (0.05 M), sodium citrate (0.05 M), sodium dihydrogen phosphate (0.2 M), disodium hydrogen phosphate (0.2 M), Tris (0.1 M), hydrochloric acid (0.1 M), sodium bicarbonate (0.05 M) and sodium hydroxide (0.05 M) were prepared by dissolving the respective analytes in water. Buffer solutions with pH of 3, 4 and 5 were prepared by adjusting the ratio of citric acid and sodium citrate standard stock solutions to 93:7, 65.5:34.5 and 41:59, respectively. Buffer solutions with pH of 6, 7 and 8 were prepared by adjusting the ratio of sodium dihydrogen phosphate and disodium hydrogen phosphate stock solutions to 87.7:12.3, 39:61 and 5.3:94.7 per 100 mL, respectively. The pH 9.0 buffer solution contained 5.6 mL of hydrochloric acid and 50.0 mL of Tris standard stock solution per 100 mL water. The pH 10 buffer solution contained 50.0 mL of sodium bicarbonate and 10.7 mL of sodium hydroxide standard stock solutions per 100 mL. The pH 11 buffer solution contained 50.0 mL of sodium bicarbonate and 22.7 mL of sodium hydroxide standard stock solutions per 100 mL water.

### Preparation of Cl^−^ standard solution

A standard stock solution (1 M) of potassium chloride was prepared by dissolving the analyte in water. Standard solutions of potassium chloride with a series of concentrations in the range of 1 × 10^−6^ M to 0.1 M were prepared by diluting the stock solution with water.

### Preparation of histidine, phosphoric acid, sodium hydroxide, hydrochloric acid and Aluminum ions standard solutions

Standard stock solutions of histidine (1 M), phosphoric acid (1 M) and sodium hydroxide (1 M) were prepared by dissolving the respective analytes in water. Standard solutions of histidine, phosphoric acid and sodium hydroxide (0.001 M) were all prepared by diluting the corresponding stock solution with water. The sodium hydroxide and hydrochloric acid solutions were standardized with primary standard solutions of potassium hydrogen phthalate and anhydrous sodium carbonate, respectively. Standard stock solution of aluminum ions (0.1 M) were prepared by dissolving analytes in water, Standard solutions of aluminum ions were all prepared by diluting the corresponding stock solution with TISAB buffer solution.

## Additional Information

**How to cite this article:** Wang, C. *et al*. Integrated multi-ISE arrays with improved sensitivity, accuracy and precision. *Sci. Rep.* **7**, 44771; doi: 10.1038/srep44771 (2017).

**Publisher's note:** Springer Nature remains neutral with regard to jurisdictional claims in published maps and institutional affiliations.

## Supplementary Material

Supplementary Information

## Figures and Tables

**Table 1:** The comparison of the relative error of pH obtained by EIMES-10 glass pH electrodes and a single glass pH electrode.

Endpoint titration	Theoretical Value	EIMES-10 pH electrodes	Relative error %	Single pH electrode	Relative error %
ep1	5.267	5.283	0.304	5.39	2.33
ep2	8.845	8.859	0.158	8.94	1.10

The endpoint pH values of the titration are calculated according to the H_3_PO_4_ titration curve obtained by titrating with standard NaOH solution.
