# Supplementary material for: Integrated multi-ISE arrays with improved sensitivity, accuracy and precision
Source: Sci Rep. 2017 Mar 17;7:44771. doi: 10.1038/srep44771 (PMC5356001; doi:10.1038/srep44771)
Supplement: Supplementary Information [file srep44771-s1.doc]

Supplementary information

# Integrated multi-ISE arrays with improved sensitivity, accuracy and precision

Chunling Wang^1^, Hongyan Yuan^1^, Zhijuan Duan^2^, Dan Xiao^1,2,*^

^1^College of Chemical Engineering, Sichuan University, 29 Wangjiang Road, Chengdu 610064, People’s Republic of China

^2^College of Chemistry, Sichuan University, 29 Wangjiang Road, Chengdu 610064, People’s Republic of China

**This document provides extra information including:**

Supplementary Figure S1: The demonstration of the phase-shift filter.

Supplementary Figure S2: The design of the multi-electrodes circuit.

Supplementary Figure S3: The circuit board of electronic integrated multi-electrodes system (EIMES).

Supplementary Figure S4: The picture of 30 lab-made chlorine ion electrodes.

Supplementary Equation: The derivation of relative error transfer formula of Equation 2 in the manuscript.


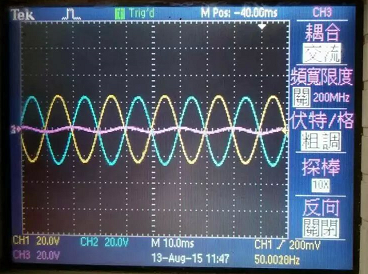


**Supplementary Figure S1:** The demonstration of the phase-shift filter.

The green curve is the 50 Hz original sine signal, the yellow curve is the signal after 180° phase-shift circuit. The pink curve is the output signal from the two-input adder.

The phase-shift filter is an effective method to separate weak Nernstian potential signal from relatively strong interference background. Although the low pass filter can remove some interference signal, but it is not the best choice. Sometimes, it can affect the measurement of the varying useful signals, if the potential difference is small which corresponding change is probably also low frequency signal. For a specific periodical noise, the phase-shift filter can removes it without effects the other signal component. The main component of the interference signal is induction from alternative current of the power supply system which is 50 Hz (green curve in Supplementary Figure S1). After the phase-shift filter, a periodical signal around 50 Hz will be shifted 180º phase (yellow curve in Supplementary Figure S1). By adding an inversed phase periodical signal, the output is almost zero (pink curve in Supplementary Figure S1).


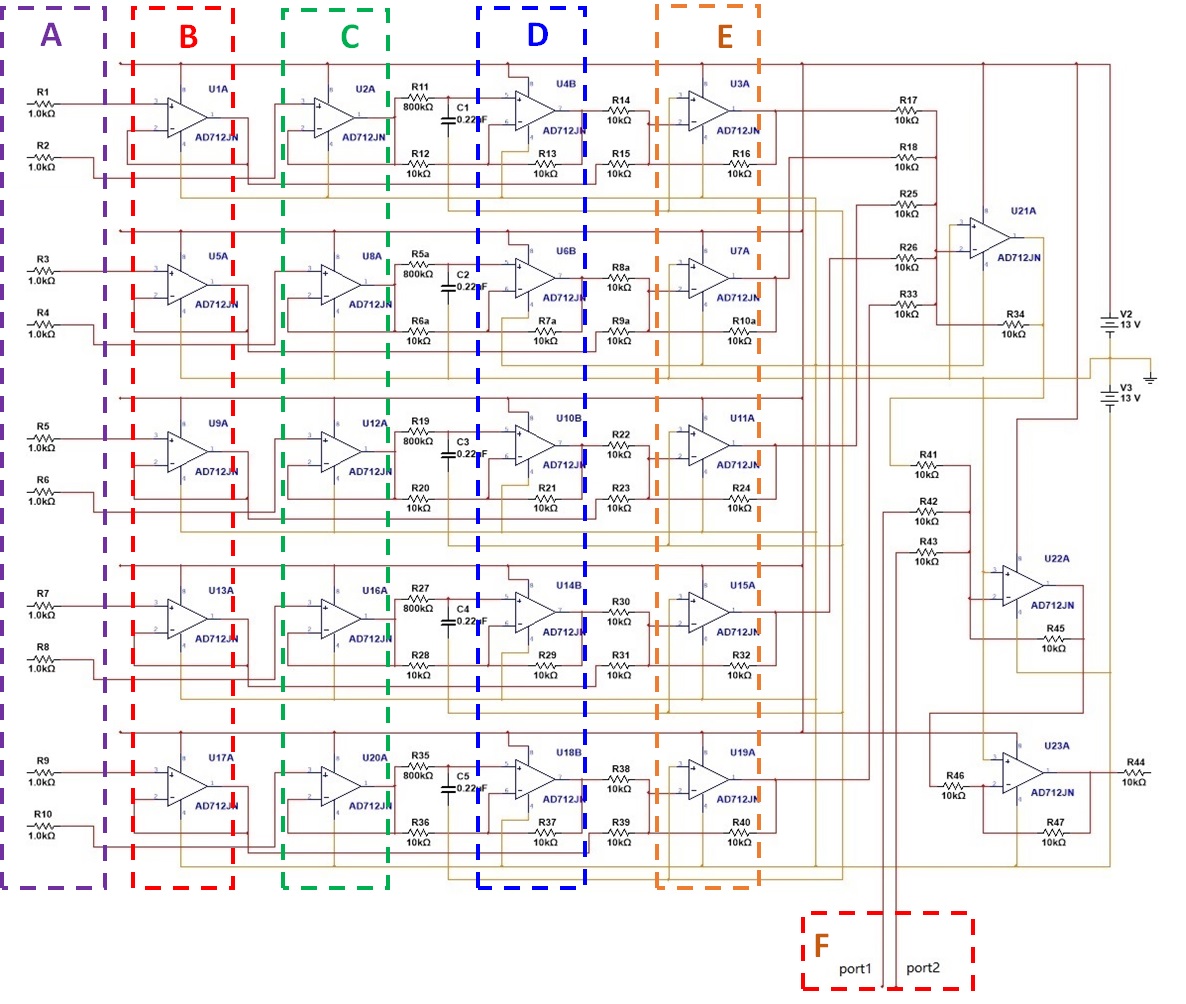


**Supplementary Figure S2:** The design figure of the multi-electrodes circuit.

All the operational amplifiers in Supplementary Figure S2 are AD712JN. The figure is one third of the whole 30 electrodes circuit. Ten electrodes input through the resistors R1 - R10 **(A)**. The first **(B)** and second **(C)** columns from the left of operational amplifiers are the voltage follower. The third column **(D)** of operational amplifiers are the phase-shifter. The fourth column **(E)** of operational amplifiers are an inversed phase adder for two inputs. Five pairs of inputs are sent to the adder U21A. The output from U21A and from other two same structure circuit boards are send to another adder U22A. The output signal from other circuit board connect this board through port1 or port2 on the bottom of the figure **(F)**. Then the sum of 30 channels is inversed through an inversed amplifier U23A (1:1) and recorded by a voltage-meter.


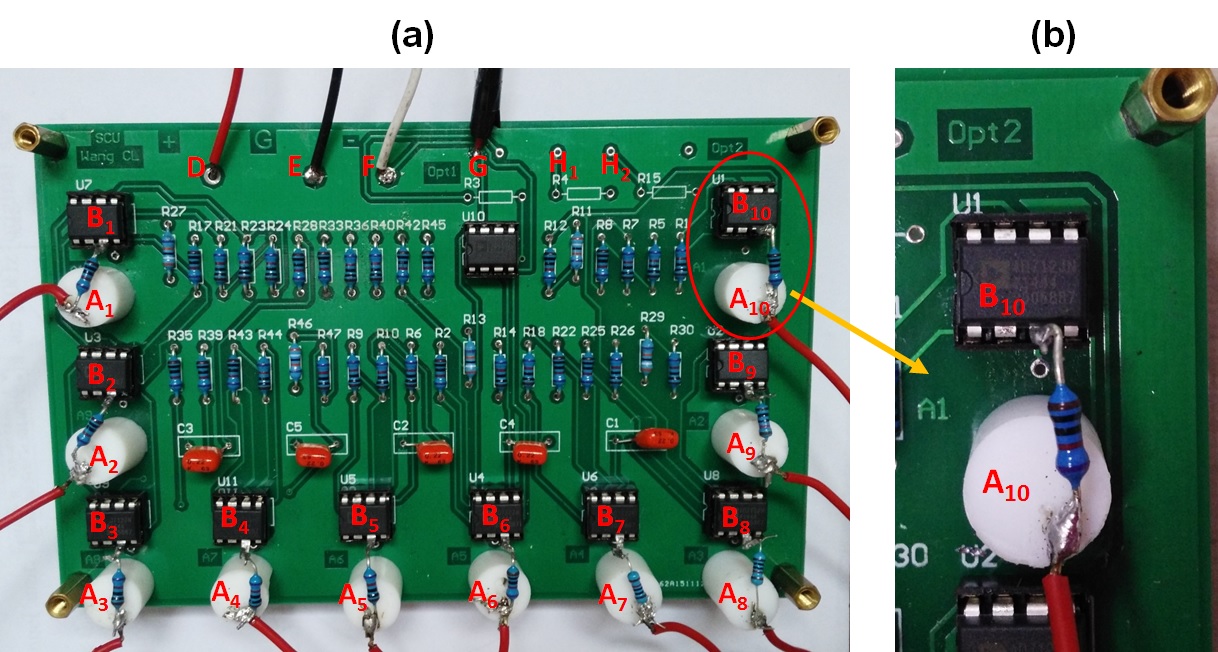


**Supplementary Figure S3:** The circuit board of electronic integrated 10-electrodes system (a). The partial enlarges view of signal input terminals of ISEs and operational amplifiers (b).

A_1_-A_10_. The signal input terminals of ISEs. Copper conductor cable as the signal transmission line. In the partial enlarges view (Supplementary Figure S3 (b)), the signal input terminals of the operational amplifiers with a resistors are raised on a white base, and connecting signal input come from electrode. Here, the white cylinder is a polytetrafluoroethylene base. The common resistance for polytetrafluoroethylene is on the order of 10^12^, which is larger than that of glass pH electrode (on the order of 10^9^), and for circuit board is on the order of 10^6^. So, the [high](javascript:void(0);) [frequency](javascript:void(0);) alternating current [signal](javascript:void(0);) can be transferred to the ground through the circuit board to decreases the stray capacity and increases the input impedance of the induced noise from the surrounding environment.

B_1_-B_10_. The operational amplifiers corresponding to the U1A to U20A in Supplementary Figure S2

C. The adder corresponding to U21A and U22A in Supplementary Figure S2.

D. The positive power connection.

E. Ground connection.

F. The negative power connection.

G. The total signal output terminals of 10 ISEs.

H_1_, H_2_. Signal input terminals from other circuit boards.


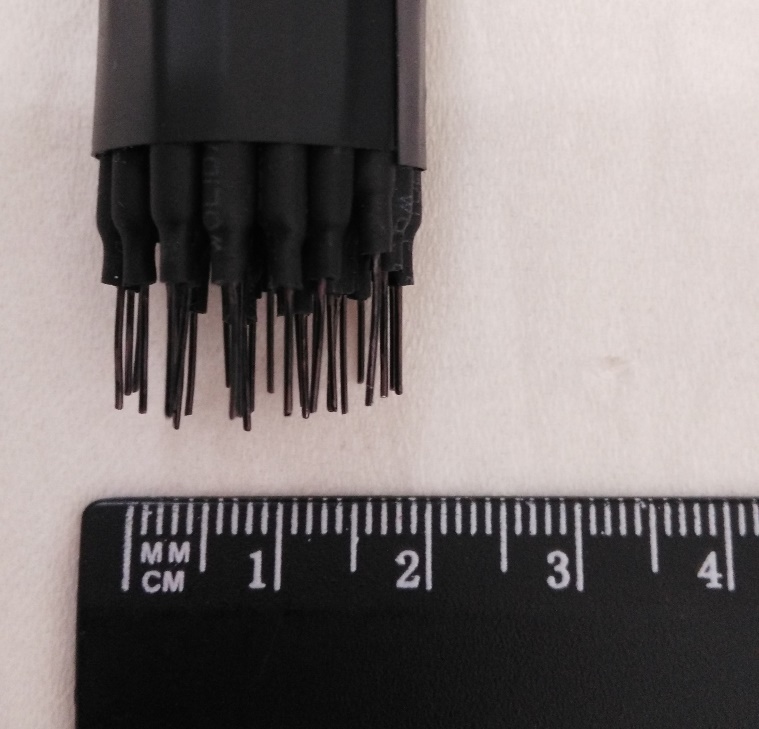


**Supplementary Figure S4: The picture of 30 chlorine ion electrodes.**

Chlorine ion electrodes were prepared by anodizing 0.5 mm diameter silver wires in diluted HCl aqueous solution.

**Supplementary Equation**

We present the derivation of relative error transfer formula (Equation 2 in manuscript) as follows.

The Nernstian equatin is:

$$\text{E}=\text{E}^{0}\pm\text{S}\times\text{ lg C}$$

(1)

where, S is the Nernstian response slope, C represents for the activity of the ion. There is:

$$\text{C}=10^{\pm\frac{\text{(E}^{0}-\text{E)}}{\text{S}}}$$

(2)

According to the rule of the error transfer formula, the absolute error of C caused E is:

$$\text{ΔC}=\frac{\text{dC}}{\text{dE}}\text{ΔE}=10^{\pm\frac{\text{(E}^{0}-\text{E)}}{\text{S}}}\times\text{ln10}\times\pm\frac{1}{\text{S}}\times\text{ΔE}=\pm2.303\times\frac{\text{C}}{\text{S}}\times\text{ΔE}$$

(3)

So, the relative concentration error of C is:

$$\frac{\text{ΔC}}{\text{C}}=\pm\frac{\text{2.303ΔE}}{\text{S}}$$

(4)
